# Multifocal Tuberculous Dactylitis Involving the Hand and Foot in a Pre-adolescent Patient: A Case Report

**DOI:** 10.7759/cureus.110156

**Published:** 2026-06-03

**Authors:** Oram R Phani, Uday K Sahu, Abhipsa Singh, Amit R Rup, Sunita Mahapatra, Swarnalata Das, Nirmal K Mohakud

**Affiliations:** 1 Pediatrics, Kalinga Institute of Medical Sciences, Bhubaneswar, IND; 2 Pediatrics, Kalinga Institute of Medical Scinces, Bhubaneswar, IND; 3 Pediatrics, Kalinga Institute of Medical Sciences, Bhubaneshwar, IND; 4 Pediatric Medicine, Kalinga Institute of Medical Sciences, Bhubaneswar, IND

**Keywords:** extrapulmonary tuberculosis, lytic bone lesions, pediatric osteomyelitis, spina ventosa, tuberculous dactylitis

## Abstract

While tuberculous (TB) dactylitis (spina ventosa) is traditionally recognized as an uncommon extrapulmonary manifestation in early childhood, its presentation in pre-adolescents remains exceptionally rare and diagnostically challenging. We report an unusual case in an 11-year-old boy who presented with a two-month history of insidious, painful, and progressive swelling of the right index finger and right foot. Initial antibiotic treatment at a local facility was unsuccessful. Physical examination revealed localized tenderness and restricted joint mobility. Laboratory investigations showed microcytic anemia and markedly elevated inflammatory markers (erythrocyte sedimentation rate (ESR) 120 mm/hr, C-reactive protein (CRP) 56 mg/L). Radiographic imaging identified characteristic expansile lytic lesions in the proximal phalanx and first metatarsal. The diagnosis was confirmed via fine-needle aspiration cytology (FNAC), which revealed acid-fast bacilli (AFB) on Ziehl-Neelsen staining. The patient was started on a standard four-drug anti-tubercular therapy (HRZE) regimen. This case underscores the importance of considering TB dactylitis in the differential diagnosis of chronic bone swelling in older children and multifocal presentations, especially in TB-endemic regions, to prevent diagnostic delays.

## Introduction

Tuberculosis (TB) predominantly affects the lungs, but musculoskeletal involvement occurs in 1% to 5% of patients [1]. Among these, tuberculous dactylitis, an infection of the short tubular bones of the hands and feet, is exceedingly rare, accounting for only 2% to 4% of skeletal TB cases [2]. Unlike classical tuberculous osteomyelitis, which typically targets long bone metaphyses in adults [3], dactylitis almost exclusively affects children under six years of age. This predilection is driven by the rich periosteal blood supply and active hematopoietic marrow in young digits [4]. As this marrow converts to fat during adolescence, the incidence plummets, making presentations in older children highly atypical [5]. The infection spreads lymphohematogenously from a primary focus, though active concurrent pulmonary TB is frequently absent [6].

Clinically, tuberculous dactylitis presents insidiously as a firm, progressive swelling of the affected digit, often without constitutional symptoms like fever or weight loss [7]. Radiographically, it progresses from soft tissue swelling to bony expansion. The characteristic ballooned, cystic appearance of the thinned cortex is historically termed spina ventosa [4,8]. Because of its indolent nature, diagnosis is often delayed, risking severe complications such as discharging sinus tracts, secondary pyogenic infections, and premature epiphyseal destruction [8]. Consequently, it must be carefully differentiated from chronic pyogenic osteomyelitis, syphilitic dactylitis, sickle-cell hand-foot syndrome, and bone tumors [5].

Definitive diagnosis requires a high index of suspicion and is confirmed via histopathology, demonstrating caseating granulomas, coupled with microbiological isolation using Acid-Fast Bacilli (AFB) smears, cultures, or GeneXpert assays [2,6]. Management is primarily conservative, relying on a 9- to 12-month course of anti-tubercular therapy (ATT) [6,7], with surgery generally reserved for biopsies or debridement of refractory abscesses [8]. We report a rare case of multifocal tuberculous dactylitis involving both the hand and foot in an 11-year-old boy, highlighting the importance of including TB in the differential diagnosis of chronic digital swelling even outside the typical early pediatric age bracket.

## Case presentation

An 11-year-old boy presented with complaints of painful swelling of the right index finger and right foot for two months. The swelling was insidious in onset, gradually progressive, associated with a continuous, throbbing type of pain, not relieved by medication. There was no history of trauma, fever, cough, hemoptysis, or hematemesis. There was a positive history of TB contact. The child was initially taken to a local hospital and was given oral antibiotics.

**Figure 1:**
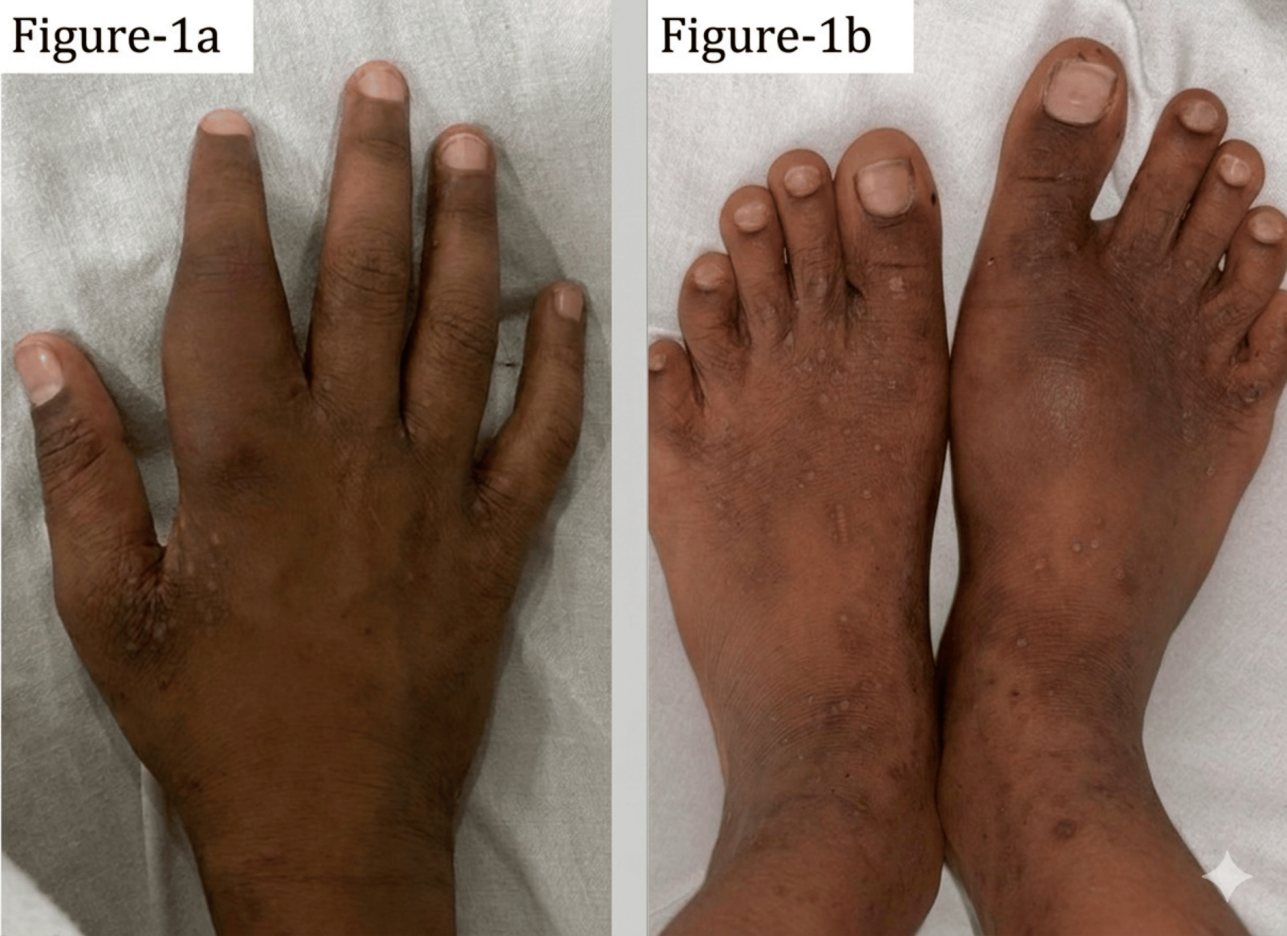
Clinical features of the right hand and left foot (a) Clinical photograph showing fusiform (spindle-shaped) swelling of the right index finger with overlying stretched skin and mild erythema. (b) Clinical photograph of the right foot demonstrating mild, diffuse swelling and soft tissue fullness, particularly over the medial aspect.

At presentation, the child was vitally stable with heart rate (HR) 88 beats per minute (bpm), respiratory rate (RR) 24/min, blood pressure (BP) 122/68 mmHg, SpO2 99 % on room air, and temperature 99.3F. His score on the Glasgow Coma Scale (GCS) was 15/15. On examination, the right hand showed a fusiform (spindle-shaped) swelling of the index finger, with mild erythema and no discharging sinus. The overlying skin appears stretched but intact. Adjacent fingers are unaffected (Figure 1a). There was also mild diffuse swelling of the right foot with tenderness and restriction of movement (Figure 1b). On general examination, the child had pallor and no cyanosis or clubbing. Systemic examination was within normal limits. An orthopedic consultation was sought at this stage to evaluate the localized musculoskeletal swelling and restricted mobility, ensuring a coordinated multidisciplinary approach to the diagnostic workup.

Initial laboratory evaluation revealed hypochromic microcytic anemia with a hemoglobin level of 8.6 g/dL. The total leukocyte count (TLC) was 8,920/µL, with a differential count showing 64.3% neutrophils and 26.2% lymphocytes. The platelet count was elevated at 4.86×105 /µL. Liver function tests (LFTs), renal function tests (RFTs), and serum electrolytes were all within normal limits (Table 1).

**Table 1:** Baseline Laboratory Investigations on Admission CBC, complete blood count; TLC, total leukocyte count; Hb, hemoglobin;  S., serum; SGOT, serum glutamic-oxaloacetic transaminase (aspartate aminotransferase); SGPT, serum glutamic-pyruvic transaminase (alanine aminotransferase); HIV, human immunodeficiency virus; HCV, hepatitis C virus; HBsAg, hepatitis B surface antigen; ESR, erythrocyte sedimentation rate; CRP, C-reactive protein. Normal reference ranges are provided for the patient's age group.

Test	Value	Normal Value
Total Leukocyte Count	8920 / µL	5000-11000 / µL
Haemoglobin (Hb)	8.6 g/dL	11.5-15 g/dL
Platelets	4.86×10^5 ^/µL	1.7-4.5 ×10^5 ^/µL
Serum Urea	15.90 mg/dL	12-42 mg/dL
Creatinine	0.31 mg/dL	0.2-0.42 mg/dL
S.Bilirubin (Total)	0.32 mg/dL	0.2-1.2 mg/dL
SGOT	26 U/L	<40 U/L
SGPT	14.90 U/L	<40 U/L
Albumin	2.97	3.5-5 gm/dL
Viral markers (HIV, HCV, HBsAg)	Non-reactive	
ESR	120 mm in 1^st^ hr	5-15 mm/hr
CRP	56.0 mg/L	<5.0 mg/L
Sickling Test	Negative	

**Figure 2:**
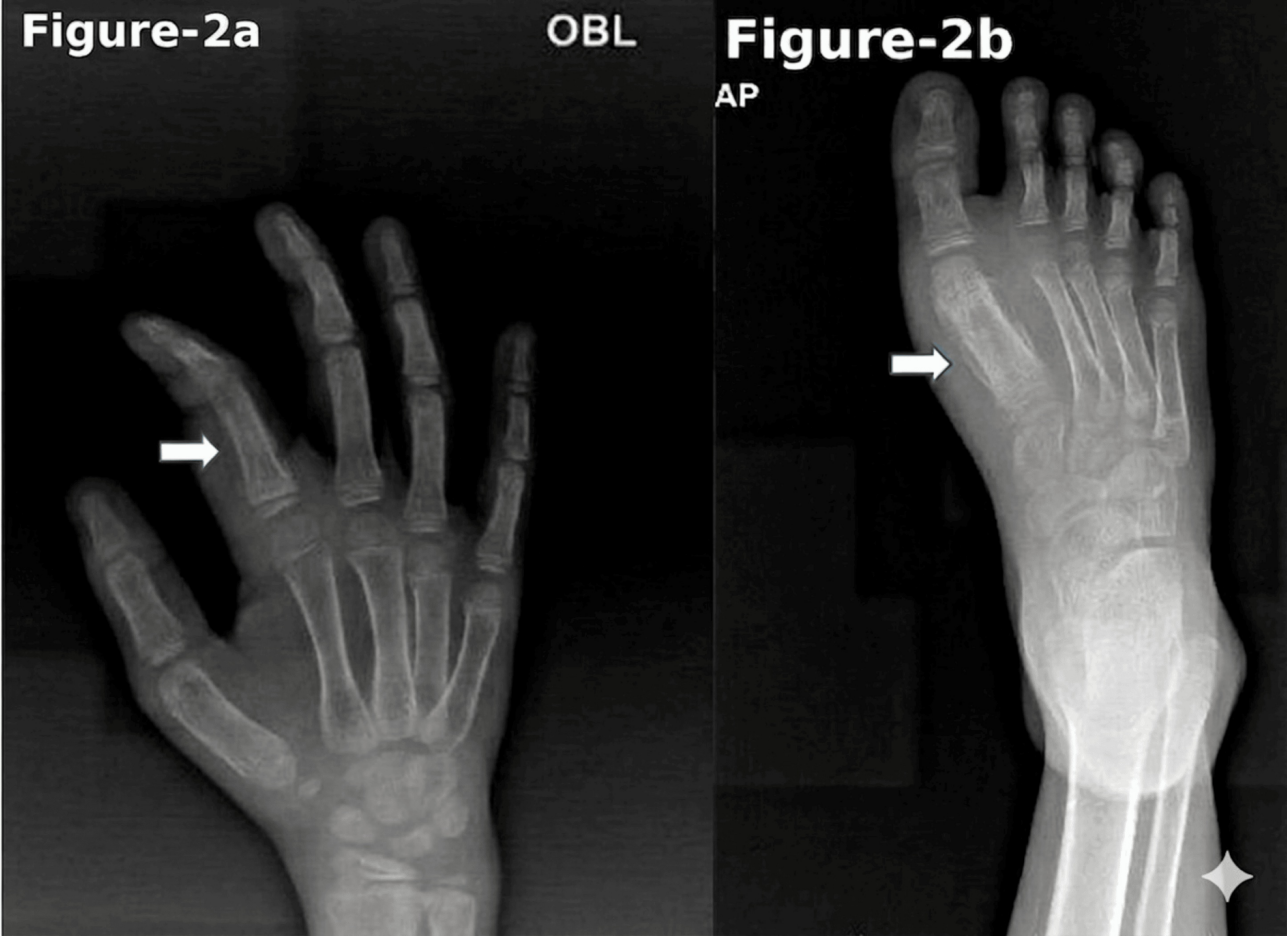
Clinical and radiographic features of the right foot (a) Plain radiograph (anteroposterior view) of the right hand revealing an expansile, "ballooned" lytic lesion (spina ventosa) of the proximal phalanx of the index finger with cortical thinning and medullary widening. (b) Oblique radiograph of the right foot revealing a well-defined, expansile lytic lesion involving the first metatarsal bone. The image shows significant cortical thinning and trabecular destruction without joint space involvement, consistent with tuberculous dactylitis.

Inflammatory markers were raised. Plain radiograph (anteroposterior view) of the right hand demonstrating an expansile, well-defined lytic lesion involving the proximal phalanx of the index finger. There is cortical thinning, medullary widening, and a "ballooned" appearance (spina ventosa) without significant periosteal reaction or sequestrum formation. The adjacent interphalangeal and metacarpophalangeal joints are preserved (Figure 2a). Oblique (OBL) view of the right foot demonstrating a similar well-defined, expansile lytic lesion in the first metatarsal bone with cortical thinning and trabecular destruction, consistent with tuberculous osteomyelitis. No joint involvement or subluxation is noted (Figure 2b).

The Mantoux test was positive with 14 mm of induration noted after 72 hours. Fine-needle aspiration cytology (FNAC) was performed, and the cytology showed sheets of intact and degenerated polymorphs, admixed with a few histiocytes and lymphocytes in a dirty necrotic background. Ziehl-Neelsen staining of the FNAC sample was positive for AFB. With the diagnosis confirmed as TB dactylitis, the child was started on anti-tubercular therapy (ATT) in accordance with the National Tuberculosis Elimination Program (NTEP) guidelines. The intensive phase comprised weight-based daily dosing of isoniazid (10 mg/kg), rifampicin (15 mg/kg), pyrazinamide (35 mg/kg), and ethambutol (20 mg/kg). Based on the patient's weight bracket, this was administered as four pediatric fixed-dose combination (FDC) dispersible tablets.

**Figure 3:**
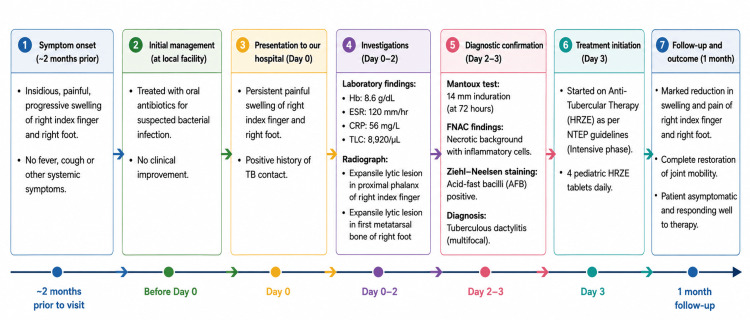
Treatment timeline of the patient The image depicts the chronological progression of symptoms, diagnostic evaluation, confirmation of multifocal tuberculous dactylitis, initiation of anti-tubercular therapy, and clinical outcome. Hb, Hemoglobin; ESR, Erythrocyte sedimentation rate; CRP, C-reactive protein; TLC, Total leukocyte count; FNAC, Fine-needle aspiration cytology, AFB, Acid-fast bacilli; HRZE, Isoniazid, rifampicin, pyrazinamide, ethambutol; NTEP, National tuberculosis elimination program. This figure was generated with assistance from ChatGPT (OpenAI, San Francisco, CA, USA; accessed May 2026) and subsequently reviewed and edited by the authors for clinical accuracy.

The patient showed a favorable clinical response to the initiation of the ATT regimen. Currently, the child’s condition has significantly improved, with a marked reduction in the swelling and pain of both the right index finger and the right foot. At the one-month follow-up, the patient remained completely asymptomatic with near-total resolution of the swelling and complete restoration of normal joint mobility. A detailed timeline summarizing the onset of symptoms, diagnostic workup, treatment course, and follow-up outcomes is presented in Figure 3.

## Discussion

An 11-year-old boy presented with a two-month history of insidious, painful, and progressive swelling of the right index finger and foot. Despite initial antibiotic treatment, the continuous throbbing pain persisted, though he exhibited no systemic symptoms like fever or cough. Notably, the patient had a positive history of TB contact, prompting further specialized clinical evaluation. Tuberculous dactylitis is a rare form of skeletal TB, accounting for a small fraction of extrapulmonary TB cases. It usually occurs in young children, and its occurrence in older children, as seen in our case, is relatively rare [9]. The hand bones are more commonly involved than those of the feet [10]. The development of such atypical, multifocal extrapulmonary manifestations relies heavily on the pathogen's ability to evade host defenses and disseminate hematogenously. The persistence of Mycobacterium tuberculosis in host tissues is driven by complex virulence factors; for instance, the PE11 gene has been shown to play a pivotal role in modulating host-pathogen interactions and facilitating prolonged bacterial survival [11]. Driven by these persistence mechanisms, the infection typically spreads through hematogenous dissemination from a primary focus, which may not always be clinically evident. In many cases, including ours, chest radiographs may be normal, with no active pulmonary disease [3].

The term "spina ventosa" is derived from the Latin spina (thorn) and ventosa (wind-filled), describing the radiographic appearance of "ballooned" bone with thinned cortices [12]. The peculiar predilection for short tubular bones in children is attributed to the presence of hematopoietic marrow in these bones and a lush blood supply through the nutrient artery [13]. As the child matures, the hematopoietic marrow is replaced by fatty marrow, and the nutrient canals gradually become obliterated, which explains why the incidence decreases significantly after age six. In older children and pre-adolescents, like the case presented here, the presentation often involves multiple sites (multifocal) due to a higher bacillary load or relative immunosuppression, though our patient was otherwise immunocompetent [14].

Clinically, patients present with gradually progressive swelling of the affected digit, often associated with mild to moderate pain. Constitutional symptoms such as fever, weight loss, or cough may be absent, making diagnosis challenging. In our patient, the swelling was insidious and progressive, with minimal systemic symptoms, which is consistent with previous reports. Laboratory findings are usually non-specific. Raised ESR and CRP are common, reflecting chronic inflammation. The Mantoux test is often positive, supporting the diagnosis in endemic regions. Definitive diagnosis requires microbiological or cytological confirmation. In our case, FNAC showed a necrotic background with inflammatory cells, and Ziehl-Neelsen (ZN) staining confirmed the presence of acid-fast bacilli, which is considered diagnostic [15]. Radiological findings typically show cystic expansion of the bone with cortical thinning and minimal periosteal reaction, resulting in the classical spina ventosa appearance [16]. The different radiological features of the condition have been grouped into three stages: Stage 1 (soft-tissue swelling and no bony changes), Stage 2 (bony expansion), and Stage 3 (destruction) [17]. In our case, the patient had bony expansion (Stage 2). Periosteal layering or thickening is generally not seen, and sequestration seldom occurs; the natural course may result in healing accompanied by shortening of the involved bone and deformity of the neighboring joint [9]. Lack of significant systemic symptoms often poses a challenge in diagnosing, leading to inappropriate treatment, complications, and delayed recovery [17]. Complications of TB dactylitis include bone destruction and shortening leading to permanent joint and bone deformities, pathological fractures, abscess and sinus formation, secondary local infections, and disseminated tuberculosis [3]. 

A broad range of conditions must be considered to avoid diagnostic delay. Pyogenic osteomyelitis usually presents with an acute onset, high-grade fever, and marked leukocytosis, whereas TB dactylitis is more insidious and chronic [13]. Sickle cell dactylitis (hand-foot syndrome) is a common differential in early childhood; however, it is typically associated with vaso-occlusive crises, occurs in patients under two years of age, and shows rapid resolution within weeks [15]. Sarcoidosis can involve the small bones of the hand (Jüngling’s disease) but often presents with characteristic lattice-like rarefaction on X-rays and systemic involvement [3]. Finally, enchondromas or osteoid osteomas may mimic the expansile appearance but typically lack the associated inflammatory markers (ESR/CRP) and systemic "dirt necrotic" cytology found in TB.

Management primarily consists of anti-tubercular therapy (ATT) in accordance with national guidelines such as the National Tuberculosis Elimination Program (NTEP). Surgical intervention is rarely required and is reserved for complications or diagnostic uncertainty. Curettage of the cavity has been recommended for avascular lesions [2]. Early diagnosis and prompt initiation of anti-tubercular therapy are associated with favorable outcomes, including resolution of symptoms and reduced risk of deformities and long-term complications, with most children demonstrating good clinical and radiological healing over time [8,12]. The possibility of multidrug-resistant TB needs to be considered in patients not responding to treatment.

Following the initiation of the NTEP-recommended ATT regimen, the patient demonstrated a prompt clinical response. At the most recent follow-up, the child’s general condition has significantly improved. There is a marked reduction in the soft-tissue swelling of both the right index finger and the right foot, with a concomitant decrease in localized pain and improved range of motion. This confirms the efficacy of the four-drug intensive phase in resolving musculoskeletal tuberculous lesions. Our case emphasizes the importance of maintaining a high index of suspicion for TB in chronic swelling of small bones, even in the absence of pulmonary involvement or classical systemic features.

## Conclusions

Tuberculous dactylitis is a rare form of skeletal tuberculosis often presenting with non-specific symptoms, leading to delayed diagnosis. This case highlights that TB should be considered in any child presenting with chronic swelling of the digits, particularly in TB-endemic regions. Early diagnosis using cytology and microbiological confirmation, followed by early initiation of ATT, can lead to good clinical outcomes and prevent long-term complications like deformities.
